# Geriatric Nutritional Risk Index (GNRI) and Survival in Pancreatic Cancer: A Retrospective Study

**DOI:** 10.3390/nu17030509

**Published:** 2025-01-30

**Authors:** Christina Grinstead, Saunjoo L. Yoon

**Affiliations:** Department of Biobehavioral Nursing Science, College of Nursing, University of Florida, 1225 Center Drive, P.O. Box 100187, Gainesville, FL 32610-0187, USA; cmg728@ufl.edu

**Keywords:** pancreatic cancer, nutritional status, survival, Geriatric Nutritional Risk Index, weight loss, cachexia

## Abstract

Introduction: Malnutrition is a major contributor to poor treatment and survival outcomes in pancreatic cancer, yet nutritional assessment is not standardized or consistently implemented in the care of oncology patients. The Geriatric Nutritional Risk Index (GNRI), calculated from serum albumin and body weight, may be useful as a practical tool for identifying patients at risk of poor nutritional status. *Purpose*: To provide a preliminary analysis using a limited selection of variables to examine the association of the GNRI at diagnosis and the GNRI change over time with overall survival in patients with pancreatic cancer. Methods: This retrospective study included 314 patients aged ≥18 years with pancreatic cancer. The GNRI was calculated at diagnosis and ≥30 days later. Patients were categorized by the GNRI at diagnosis (no risk >98, any risk ≤98) and change in the GNRI over time (no change/increase, mild decrease, and severe decrease). Additional variables included were demographics and stage. Comparative analysis included *t*-tests, chi-square tests, and ANOVA. Survival was analyzed using Kaplan–Meier curves, log-rank tests, and Cox proportional hazards modeling. Results: Median survival was significantly decreased in patients in the *any-nutritional-risk* group compared to the *no-nutritional-risk* group at diagnosis (442 vs. 1105 days), and those experiencing *severe decreases* in the GNRI scores compared *to mild decreases* and *no change or increases* (372.5 vs. 712 vs. 1791 days), respectively. Survival analysis stratified by the GNRI at diagnosis shows that both mild (HR 2.19, 95%, and CI 1.46–3.30) and severe decreases (HR 4.04, 95%, and CI 2.64–6.18) in the GNRI scores were independently associated with decreased survival versus *no change or increase* in the GNRI group after controlling for stage. Log-rank tests also show patients with *any nutritional risk* at diagnosis had significantly lower survival than those with *no nutritional risk* (*p* = 0.00052). Conclusions: Lower GNRI scores showing greater nutritional risk at diagnosis and decreasing GNRI scores over time were predictors of decreased survival in pancreatic cancer. Our findings indicate that the GNRI may be valid and effective for the early identification of patients with a high nutritional risk who require nutritional interventions to improve outcomes in pancreatic cancer. However, more research is needed using larger samples and a greater variety of variables to confirm the presence and strength of this relationship, examine the effect of patient factors known to be associated with survival and nutrition, and explore potential influential confounders.

## 1. Introduction

Pancreatic cancer (PC) remains one of the most lethal malignancies, with a 5-year relative survival rate of only 13% after diagnosis [1]. Poor survival and other adverse outcomes, such as decreased quality of life and suboptimal treatment outcomes, are often exacerbated by nutritional deficits, causing health disequilibrium commonly experienced in this patient population [2,3,4,5,6].

Patients with pancreatic cancer suffer profound and involuntary weight loss, anorexia, and malnutrition stemming from the catabolic effects of the tumor itself as well as the consequences of cancer treatments (e.g., surgery, chemotherapy, and radiation) [2,3,6,7]. The development of cancer cachexia, a severe wasting syndrome characterized by systemic inflammation, negative protein and energy balance, and depletion of skeletal muscle and adipose tissue, affects over 70% of patients with pancreatic cancer [3,8,9]. Cachexia is a complex metabolic condition driven by tumor-derived factors that promote muscle wasting, adipose tissue browning, and anorexia [8,9]. The dramatic muscle and weight loss associated with cachexia [9], along with the many other nutrition-related deficits common in PC, lead to higher treatment toxicity, longer hospitalizations, reduced quality of life, and worse survival outcomes [3,4,5,6,10].

Nutritional status has received increased attention as a critical factor influencing survival; however, there are no gold standard or universal nutritional assessment methods for the early identification of nutritional risk in patients with pancreatic cancer [3,11,12], and current practices are in need of methodological improvement [3,5,6,7,10,11,12]. Given the difficulty of maintaining adequate nutritional status in patients with pancreatic cancer due to the effects of both the cancer itself and its treatments, there is a pressing need for early detection of nutritional risk using practical tools [2,3,6,10,12] that are sensitive [6] and able to work effectively for real-life patients and in the current healthcare system [13,14].

While computed tomography (CT) or magnetic resonance imaging (MRI) are objective methods to assess nutritional status [15] and cachexia in oncology [2,3], these methods are costly and time-consuming, requiring specialized equipment [2,16]. Blood-based biomarkers (e.g., C-reactive protein and albumin) have been studied as potential indicators of nutritional deficits and cachexia, but no single biomarker has proven sufficiently accurate or reliable on its own [3,11]. Several tools exist to strengthen these weaknesses by combining these single markers to create a comprehensive scoring system [17,18,19] or the American Society of Anesthesiologists’ score [20]. In contrast, other studies reported conflict findings indicating decreased effectiveness of the GNRI in identifying patients at greater risk of negative outcomes for certain patient subgroups, for example, those who have had pancreatic resection surgery [21,22], using multiple tools. Nevertheless, the GNRI may benefit clinical practice [3,11,13].

The Geriatric Nutritional Risk Index (GNRI), originating from the Nutritional Risk Index (NRI), is a tool that uses a combination of markers to assess nutritional risk specifically in older adults for whom detailed information on weight and recent weight loss may be unknown [23]. It has shown potential in predicting survival in cancer populations [24,25]. The GNRI uses two routinely available parameters in the clinical setting, serum albumin and body weight, to calculate nutritional risk [23], providing an accessible and practical approach to nutritional evaluation. The GNRI has shown prognostic value for predicting mortality and other adverse outcomes in various diseases beyond the geriatric population, including heart failure, chronic kidney disease requiring dialysis, and various cancers [22,24,25,26,27,28]. Its simplicity allows for easy calculation and understanding, increasing the potential for use to identify those needing nutritional support.

While there has been promising research in recent years showing a significant association between the GNRI and survival in PC [25,28,29,30,31,32], studies incorporating additional covariates and potential confounders have shown mixed results, indicating that the relationship between the GNRI and survival in PC may differ based on factors such as age [33], comorbidities [20], and treatment modality [31]. Other studies have shown that the GNRI is associated with clinical characteristics, including tumor location [19] and age, as well as blood-based biomarkers (e.g., carcinoembryonic antigen (CEA) and hemoglobin) [32]. More research is needed to determine the relationships between the GNRI, nutritional status, and outcomes in PC patients [24] and the influence of factors on the GNRI in PC. Despite the increased number of studies on the GNRI and PC, the sample sizes and scopes of the studies were limited. Most studies have been retrospective and used small sample sizes [19,21,25,29,31,33]. Most studies used only cross-sectional measures of the GNRI score at diagnosis or prior to treatment or surgery [19,20,21,25,28,29,31,32,33]. Longitudinal changes in the GNRI scores may provide us with more prognostic and predictive insights.

In summary, as patients with PC are often challenged to maintain optimal nutritional status and weight for positive treatment outcomes, a nutritional risk assessment using the GNRI may assist in identifying those most vulnerable to nutritional decline, leading to premature mortality. Determining whether the GNRI and longitudinal changes in the GNRI scores are associated with survival could provide essential information in caring for patients with PC for nutritional monitoring and tailored, personalized interventions of this high-risk population where maintaining nutritional status remains of great importance.

This pilot study aims to examine potential relationships between changes in the GNRI over time, the GNRI at diagnosis, and overall survival in patients with PC. We hypothesized that lower baseline GNRI scores and decreasing GNRI scores over time would be associated with shorter survival, even after controlling for the patient characteristics. These results may show the importance of changes in the GNRI scores over time and the baseline GNRI status on survival outcomes in future research efforts.

## 2. Materials and Methods

### 2.1. Data Source

The de-identified data were obtained from the Integrated Data Repository (IDR), Clinical and Translational Science Institute (CTSI) at the University of Florida (UF), U.S.A. As the honest broker and not part of the research team, the IDR team retrieved the data from the electronic health record system. The study has been approved by the UF Institutional Review Board (IRB) (IRB#202001897).

### 2.2. Samples

**Figure 1:**
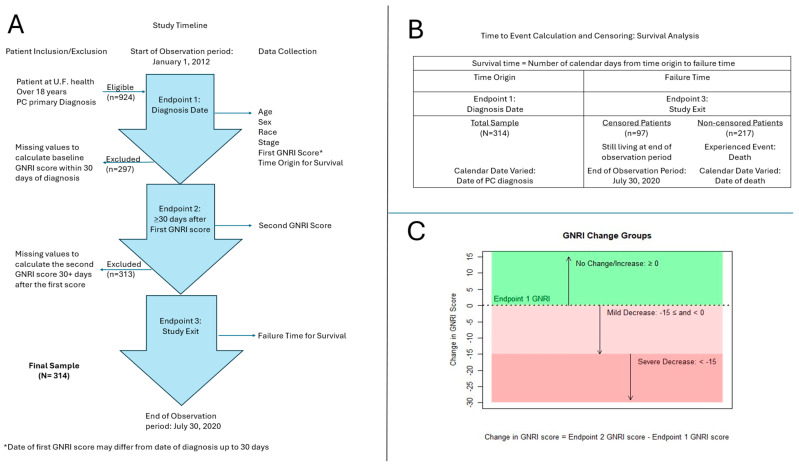
Illustration of methods and data collection processes. (**A**) Study timeline showing observation period, data collection timepoints, and sample selection process, including patient inclusion and exclusion criteria. (**B**) Calculation of survival time. (**C**) Description of GNRI change groups based on endpoint 1 GNRI score.

De-identified data from 924 patients with pancreatic cancer were eligible for the study. Inclusion criteria were patients who (1) visited UF Health between 1 January 2012 and 31 July 2020, (2) were 18 years of age and older at the time of data collection, (3) had a primary cancer diagnosis of pancreatic cancer, and (4) had two data points of weight and albumin levels within 30 days of diagnosis and at least 30 days following the initial measurement to calculate the GNRI scores. Data from 610 patients were excluded because of (1) no measurement points to calculate a GNRI score within 30 days of the diagnosis date (*n* = 297) and (2) no measurement points to calculate the second GNRI score that was at least 30 days after the first GNRI score was measured (*n* = 313). The final analysis for this retrospective study includes data from 314 patients. Figure 1A presents a detailed description of the sample selection process.

### 2.3. Measurements

Variables included demographics, cancer stage, the GNRI scores at diagnosis, changes in the GNRI scores, and survival days.

#### 2.3.1. Data Collection Endpoints

Of the data that met the inclusion/exclusion criteria, variables for the GNRI were measured at three endpoints based on the data collected, and demographic data were collected only at the baseline. Figure 1A describes the three endpoints and corresponding data collection.

Endpoint 1 was defined at the time of cancer diagnosis, which considered the time origin for survival. Data collected at endpoint 1 included age, sex, race, and cancer stage. The calendar date of the time origin for survival at endpoint 1 varied depending on the date of cancer diagnosis for each patient. The first GNRI scores for endpoint 1 were data on weight and albumin levels collected at diagnosis with up to 3 days difference between weight measures and the albumin levels due to the variability in the data collection dates. For this same reason, the date of the first GNRI measurement may differ up to 30 days from the date of diagnosis.

Endpoint 2 was defined as the date of the latest available GNRI scores, which was at least 30 days after the first GNRI score, allowing for a meaningful difference. The calendar date and number of days between the first and second GNRI scores varied among patients’ data and were subject to data availability.

Endpoint 3 was defined as the time of study exit and the failure time for survival. With the use of survival analysis, censoring of patients was performed based on endpoint 3. This endpoint did not require further follow-up data collection as the mortality status of each patient included in the study was confirmed through the end of the observation period. Patients who confirmed death within the time of the observation period were non-censored and had a failure time corresponding with their date of death (*n* = 217). Patients confirmed to remain living at the end of the observation period were censored and assigned a failure time of the same date as the end of the observation period of 31 July 2020 (*n* = 97).

#### 2.3.2. Demographic and Clinicopathologic Characteristics

Due to the sample size, the following variables were combined into two categories for analysis: The cancer stage was categorized into early (stages 1 and 2) and late stages (stages 3 and 4). Race was categorized into White and all Other. Age was analyzed as a continuous variable.

#### 2.3.3. Survival

Survival was defined as the number of days from diagnosis to the date of death. It corresponds to endpoint 1, indicating the time origin for survival, and endpoint 3, indicating the failure time for survival. Patients alive as of 31 July 2020 were considered living as the last data collection date, and the date of death was set for this same date. A censoring variable was included in the survival analysis, with death as the event of interest. See Figure 1B for a summary of how survival time was calculated for this study.

#### 2.3.4. The Geriatric Nutritional Risk Index (GNRI)

The GNRI is calculated by first using the Lorentz formula to determine the ideal weight based on sex as follows [23]:

Men: H − 100 − [(H − 150)/4](1)

Women: H − 100 − [(H − 150)/2.5],(2)

Then, the ideal weight is substituted into the following equation to determine the GNRI score, with WLo = 1 when the weight exceeds the ideal weight [23]:

[1.489 × albumin (g/L)] + [41.7 × (weight/WLo)](3)

This equation was used to calculate two variables within the study based on the first GNRI score using data collected at endpoint 1 and the second GNRI score using data collected at endpoint 2 (see Figure 1A). The two variables created using the GNRI equation were (1) the GNRI score at diagnosis and (2) the change in the GNRI score over time. The GNRI score at diagnosis, or the score calculated using data collected at timepoint 1, was used to categorize patients into two groups (*no nutritional risk*: GNRI > 98; *any nutritional risk*: GNRI ≤ 98) [23].

The change in the GNRI score variable was determined using both the GNRI score at diagnosis (endpoint 1) and a second GNRI score calculated using the latest available data at least 30 days after the first score for each patient included (endpoint 2). The number of days in between the two measurements was varied between patients. A change in the GNRI score was determined by taking the difference by point between these two GNRI scores. This value was then used to categorize patients into three groups: (1) no change or increase: GNRI change ≥ 0, (2) mild decrease: −15 ≤ GNRI change < 0, and (3) severe decrease: GNRI change < −15 (see Figure 1C).

### 2.4. Statistical Analysis

Statistical analysis was conducted using R Studio (version 4.2.3, R Foundation for Statistical Computing: Vienna, Austria) [34].

#### 2.4.1. Comparative Analysis

Descriptive statistics were conducted for the total sample with frequencies, means, and medians, as well as the GNRI scores at diagnosis and changes in the GNRI scores over time by groups. Comparative analyses were performed using chi-square tests, *t*-tests, and ANOVA depending on the characteristics of the variables. The survival variable was not included in the comparative analysis due to censoring within the data to avoid bias. Statistical significance was set at *p* < 0.05.

#### 2.4.2. Survival Analysis

Kaplan–Meier curves and log-rank tests were conducted for each variable individually. The primary predictive variable of interest was the change in the GNRI scores. Cox regression analysis was conducted to identify the optimal model to predict survival using all available variables and included identifying violations of the proportional hazards assumption. Multiple models were compared based on significance, violation of assumptions, Akaike Information Criterion (AIC), Bayesian Information Criterion (BIC), and consideration of model parsimony, with a final model identified to most successfully predict survival. As the final model chosen included stratification of the GNRI scores at diagnosis, subgroup analysis was conducted with the other two significant variables in the final Cox model to determine if a significant relationship was present. Statistical significance was set at *p* < 0.05.

## 3. Results

### 3.1. Descriptive Statistics and Comparative Analysis

The descriptive statistics in Table 1 highlight differences based on the GNRI scores (*no nutritional risk* vs. *any nutritional risk*) at diagnosis. Patients with *any nutritional risk* had shorter median survival days (442 days vs. 1105 days), were more likely to have late-stage cancer (53.4% vs. 37.0%), and there were more patients with *no change/increases* or *mild decreases in the GNRI scores* over *time* compared (27.8% vs. 17.7% and 48.1% vs. 44.7%, respectively) to ones with *no nutritional risk*. The comparative analysis between *any-nutritional-risk* and *no-nutritional-risk* groups shows significant differences in cancer stage (*p* = 0.0056) and changes in the GNRI scores over time (*p* = 0.0168)

Comparing patients based on changes in the GNRI scores over time, the results show those with *severe decreases* in the GNRI scores had the worst median survival (372.5 days) compared to those with *mild decreases* (712 days) or *no change/increase* (1791 days). The *severe decrease* group also had the highest proportion of late-stage cancer (41%) and *no nutritional risk* at diagnosis (68%). Comparing the two groups based on *changes in the GNRI scores* over time, only the GNRI at diagnosis (*p* = 0.00293) showed statistically significant differences between the two groups, but cancer stage (*p* = 0.0775) and sex (*p* = 0.0508) did not.

### 3.2. Univariate Survival Analysis

**Figure 2:**
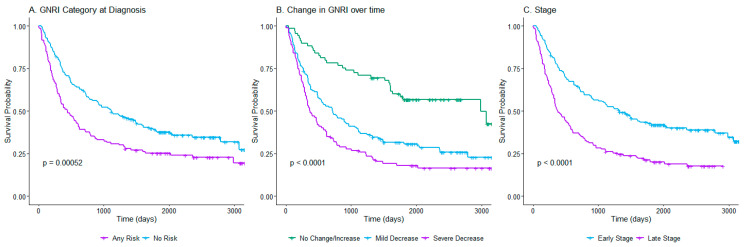
Kaplan–Meier survival curves for the variables showing statistically significant differences. (**A**) Comparing patients based on GNRI scores at the time of diagnosis categorized as any risk and no risk. (**B**) Comparison of groups based on changes in GNRI scores over time categorized as no change/increase, mild decrease, or severe decrease. (**C**) Comparison by cancer stage categorized as early or late stage.

Log-rank tests compared the survival distributions between different groups for each variable. Significant differences were found between the *any-nutritional-risk* and *no-nutritional*-*risk* groups for the GNRI at diagnosis variable (*p* = 0.00052), *changes in the GNRI scores* over time groups (*p* < 0.0001), and cancer stage (*p* < 0.0001) but not for sex (*p* = 0.3), race (*p* = 0.9), or age (*p* = 0.6). Figure 2 shows the Kaplan–Meier curves for the significant results. Patients with *no nutritional risk* at diagnosis, *no change or an increase* in the GNRI score, and at early stages had the lowest risk of death, while patients with *any nutritional risk* at diagnosis (Figure 2A), *mild decrease* or *severe decrease* in the GNRI score over time (Figure 2B), and later stages (Figure 2C) had worse survival. Additionally, those with *severe decreases* in the GNRI score over time had a higher risk of death compared to those with *mild decreases.*

### 3.3. Multivariate Survival Analysis

#### 3.3.1. Model Selection

Three Cox proportional hazard models were compared (See Table 2):

- Model 1: A model with all variables;
- Model 2: A model with non-significant variables (sex, race, and age) removed;
- Model 3: A stratified model stratified by GNRI category at diagnosis.

The first model included all available variables. The second model included only those variables that showed significance in the first model. The second model showed marginally better performance than the first but with greater parsimony. However, the GNRI scores at diagnosis violated the proportional hazards assumption in both models 1 and 2.

A third model was compared using stratification to address this violation and was found to perform best based on lower AIC (1899.65 vs. 2199.43 and 2194.38 for models 1 and 2, respectively) and BIC (1909.79 vs. 2223.09 and 2207.90 for models 1 and 2, respectively), as well as clinical interpretability, and more closely met the proportional hazards assumption compared to the other models.

In the stratified model 3, the proportional hazards assumption was still violated; however, considering that the variable of interest was the primary reason for the violation and a review of the Schoenfield residual plots showed the violation to be minor, it was determined that the model would be acceptable for the purposes of this study.

#### 3.3.2. Stratified Model Interpretation

In the stratified model, the hazard ratios shown in Table 3 indicate that patients with *a mild decrease* in the GNRI scores had a 2.194 times higher risk of death (*p* = 0.000159 and 95% CI [1.459–3.299]) compared to those with *no change/increase*, after stratifying by the GNRI category at diagnosis and controlling for cancer stage. Patients with *severe decreases* in the GNRI scores had an even higher risk of 4.038 (*p* < 0.0001 and 95% CI [2.640–6.177]). Late-stage cancer was associated with a 1.947 times higher risk of death (*p* < 0.0001 and 95% CI [1.478–2.565]) compared to early-stage cancer after controlling for changes in the GNRI scores over time and stratifying by the GNRI score at diagnosis.

#### 3.3.3. Analysis of Stratified Variable

**Figure 3:**
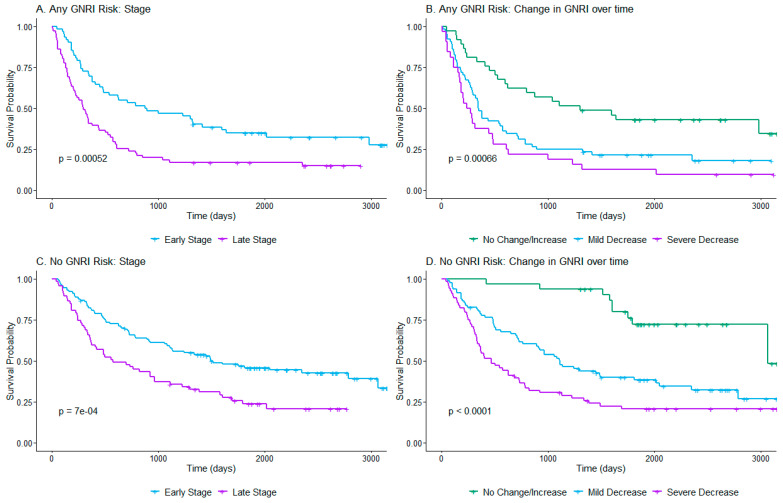
Kaplan–Meier Survival curves by GNRI category at diagnosis subgroup for Cox model variables. (**A**) Comparison of survival by stage for patients at *any nutritional risk* at diagnosis. (**B**) Comparison of survival by GNRI change over time groups for patients with *any nutritional risk* at diagnosis. (**C**) Comparison of survival by stage for patients with *no nutritional risk* at diagnosis. (**D**) Comparison of survival by change in GNRI over time for patients with *no nutritional risk* at diagnosis.

In the final Cox model, it was necessary to stratify by the GNRI category at diagnosis due to a violation of assumptions. Analysis using more appropriate methods without requiring proportional hazards shows there may still be an important relationship between the GNRI at diagnosis and the factors shown to influence survival through our chosen Cox model. The results are shown in Figure 3. For the patients at any risk based on the GNRI at diagnosis, there was a significant difference in survival between groups compared by stage (*p* = 0.00052) (Figure 3A) and changes in the GNRI scores over time (*p* = 0.00066) (Figure 3B). These same variables also showed significant differences in the patients at no risk at diagnosis (stage: *p* < 0.0001 and change in GNRI: *p* < 0.0001). Survival between groups showed similar patterns as the univariate analysis of the total sample when comparing cancer stage and changes in the GNRI scores over time groups, with early stage having more prolonged survival compared to late stage (Figure 3C), patients with no change or an increase in the GNRI score over time having the highest survival compared to those with *mild or severe decreases*, and patients with *severe decreases* in the GNRI scores having the shortest survival (Figure 3D).

## 4. Discussion

Previous studies support our findings, indicating associations between nutritional risk and mortality in solid tumors [5,35], even though conflicting results have been reported in hematologic cancers [35]. Significant associations were reported between nutritional status and quality of life, functional status, and survival in patients with PC [10,36]. Further, associations were reported between lower GNRI and decreased survival in lung and gastrointestinal cancers [22] and PC [24,28]. However, to our knowledge, this is the first study evaluating *changes in the GNRI scores*, specifically in PC, where maintaining nutritional status remains the utmost clinical challenge.

The findings of this study suggest that the GNRI scores at diagnosis and their changes over time have significant associations with survival in PC. The comparative analysis shows there to be differences between the *any-nutritional-risk* (GNRI score ≤ 98) and *no-nutritional-risk* groups (GNRI score > 98) at diagnosis for cancer stage. Changes in the GNRI scores with earlier stages and *severe decreases* in the GNRI scores over time are more common in patients at *no nutritional risk* at diagnosis. The median survival days were 442 days vs. 1105 days between the *any-nutritional-risk* and *no-nutritional-risk* groups. Our findings are consistent with other studies showing patients with lower baseline GNRI scores to have shorter survival among patients in the late stage [25] and postoperative patients with PC [28]. Other studies show conflicting results for covariates when comparing baseline GNRI score groups in pre-operative PC patients, with no difference in stage [28,32] and significant differences by sex shown [29]. Discrepancies in the findings of these studies may be due to the differences in sample population characteristics, timing of measuring the GNRI scores, and the GNRI score cutoff for group classification.

The group with a *severe decrease* in the GNRI scores had the highest proportion of patients in the *no-risk group* at diagnosis (68.0% vs. 55.9% and 46.4% for *mild decrease* and *no change/increase*, respectively), but the group showed the shortest median survival days among the three groups (372.5 days vs. 712 and 1791 for *mild decrease* and *no change/increase,* respectively). We hypothesize that a *severe decrease* in the GNRI score changes may be due to symptoms associated with PC treatments [37] and heterogeneous genetic changes in PC development [38]. However, these variables (e.g., treatment modality as neoadjuvant or adjuvant and genetic data) were not available for our analysis, which is a limitation of our study.

Differences in survival based on the GNRI scores at diagnosis with univariate analysis were consistent with the survival analysis results from recent studies looking at baseline GNRI scores in various PC populations, with lower GNRI scores being predictive of shorter survival [28,29,31,32].

In the multivariate model, patients at advanced stages had almost two times the risk of death after controlling for the GNRI score at diagnosis and change over time. These results are consistent with recent survival analyses showing that patients in later stages demonstrated decreased survival even after controlling for covariates [32]. Additionally, patients with *mild* and *severe decreases* in the GNRI scores over time had greater than two and four times the risk of death, respectively, compared to patients whose GNRI scores stayed *the same or increased* after controlling for cancer stage and the GNRI scores at diagnosis. One study reported that the GNRI scores later in the disease trajectory are stronger predictors of survival in patients with decompensated heart failure [39], while another study indicated changes in the GNRI scores corresponded with changes in other commonly accepted nutritional markers to accurately reflect changes in nutritional status over time in hemodialysis patients [40], showing support for the theory that a decline in the GNRI score and corresponding nutritional status may be a predictor of survival. The lack of comparable studies and the striking results of this study further highlight the need for more research to examine changes in the GNRI scores over time in PC patients.

Considering the limitations of stratification in the final Cox model, further analysis confirms these same patterns of survival were present in patients in both the *any-nutritional-risk* and *no-nutritional-risk groups* at diagnosis. Regardless of their GNRI scores at diagnosis, patients in both groups (*any-nutritional-risk* and *no-nutritional-risk groups)* showed significantly decreased survival for those at advanced stages and those with decreasing GNRI scores over time compared to those with stable or increasing scores. Patients with both *no nutritional risk* at diagnosis and stable (no change or increased) GNRI scores over time had the highest survival of all patient groups based on the two GNRI-related variables. Our study did not show age to be a significant predictor in either the univariate or multivariate model, but a recent study with conflicting results showed that the baseline GNRI score had a greater influence on survival in elderly patients compared to those at younger ages [33].

The prevalence of cachexia in patients with pancreatic cancer may explain the results of our study and the association between the GNRI and survival in this patient population. Cachexia is a condition driven by tumor-derived inflammatory factors, metabolic derangements, and anorexia and is significantly associated with shorter survival [41]. While the GNRI is not a direct assessment for cachexia, the GNRI may be considered valuable in identifying patients with this complex syndrome. With weight and albumin as its basis, the GNRI scores can detect patients at risk of or experiencing visceral protein depletion, weight loss, and negative protein/energy balance [42]. Recent research showed evidence of a relationship between the GNRI score and the presence of cachexia along with its associated adverse outcomes in lung and colorectal cancers [42,43]. More research is needed to determine the association of the GNRI scores with cachexia trajectory from pre-cachexia to cachexia to refractory cachexia. The GNRI may serve as a surrogate for identifying those at the highest risk of developing refractory cachexia and requiring more aggressive nutritional intervention, which is of increased necessity in pancreatic cancer, but this has not yet been confirmed.

While this study has multiple strengths, it also has several limitations, which need careful interpretation of results. Due to the nature of the retrospective study, not all the variables required for certain analyses were available. The second limitation is the exclusion of a large number of patients at endpoint 1 and endpoint 2, which may have introduced bias within the sample. A supplementary analysis was conducted to assess the bias by comparing patients who were excluded to those included within the study for the variables except for the GNRI scores (see the Supplementary Materials). Thirdly, while the first GNRI score measured at endpoint 1 was required to be within 30 days of diagnosis, the number of days in between timepoint 1 and the second GNRI score at timepoint 2 was vastly different between patients, with the only requirement being that they were over 30 days apart. Survival analysis was conducted to mitigate this limitation using the censoring variable. It was possible that the number of days between the two GNRI measurements was moderately correlated with survival (r = 0.45). These analyses reduced the risk of bias but were unable to eliminate it. Lastly, the sample population was from a single institution in the Southeast region of the United States. The generalizability of the study findings is limited to the population in a similar area.

While the limitations of this study require that results be considered suggestive of potential relationships rather than conclusive evidence, it still provides valuable information due to the significant gap in current research in which data examining changes in the GNRI scores over time and survival is lacking. This study’s findings show the potential for essential relationships between the GNRI score changes and PC survival and highlight the importance of exploring this relationship, and there is much to learn by conducting future studies using prospective designs, greater consistency and frequency of the GNRI measurements, larger sample sizes, and a more significant number of influential patient factor and suspected cofounders within the mathematical models.

## 5. Conclusions

Survival in patients with PC may be significantly associated with nutritional risk at diagnosis and its changes over time measured by the GNRI. These novel findings show that *changes in the GNRI scores* over time may have a significant influence on survival, indicated by the greater than two and four times increased risk of death in patients with *mild* and *severe decreases* in the GNRI score over time, respectively, compared to patients with stable scores even after controlling for cancer stage and the GNRI scores at diagnosis. Our findings indicate that the GNRI may be valid and effective for identifying patients at nutritional risk at diagnosis and continued monitoring of nutritional status over time. However, more research is warranted to draw definitive conclusions. The GNRI may identify PC patients with declining nutritional status early to facilitate timely and effective nutritional interventions for patients, which may increase their survival. The findings of this study may guide the design of future studies seeking to address the current research gap in examining the relationship between changes in the GNRI scores over time and survival in PC in the context of possible influential factors while utilizing increasingly robust research designs and methods, which currently are lacking.

## Figures and Tables

**Table 1:** Descriptive and comparative analysis by GNRI variables.

Variables	Total (N= 314)	GNRI at Diagnosis			Change in GNRI Over Time			
		No Risk (*n* = 181)	Any Risk (*n* = 133)	*p*-Value	None/Increase (*n* = 69)	Mild Decrease (*n* = 145)	Severe Decrease (*n* = 100)	*p*-Value
Survival (days)								
Mean	1093.8	1237	899		1671	1037.1	777.8	
Median	725	1105	442		1791	712	372.5	
Range	6–3164	44–3164	6–3154		43–3155	15–3164	6–3152	
Age (years)				0.1792				0.195
Mean	70.5	69.8	71.5		68	71.2	71.4	
Median	71	71	73		69	72	73	
Range	30–98	30–98	36–91		36–91	33–98	30–93	
Sex				0.2321				0.0508
Male	174 (55.4%)	106 (58.6%)	68 (51.1%)		33 (47.8%)	91 (62.8%)	50 (50.0%)	
Female	140 (44.6%)	75 (41.1%)	65 (48.9%)		36 (52.2%)	54 (37.2%)	50 (50.0%)	
Race				0.9748				0.9694
White	263 (83.8%)	151 (83.4%)	112 (84.2%)		58 (84.1%)	122 (84.1%)	83 (83.0%)	
All Other	51 (16.2%)	30 (16.6%)	21 (15.8%)		11 (15.9%)	23 (15.9%)	17 (17.0%)	
Cancer Stage				**0.0056**				0.07752
Early	176 (56.1%)	114 (63.0%)	62 (46.6%)		45 (65.2%)	72 (49.7%)	59 (59.0%)	
Late	138 (43.9%)	67 (37.0%)	71 (53.4%)		24 (34.8%)	73 (50.3%)	41 (41.0%)	
Diagnosis GNRI								**0.00293**
No Risk	181 (57.6%)				32 (46.4%)	81 (55.9%)	68 (68.0%)	
Any Risk	133 (42.4%)				37 (53.6%)	64 (44.1%)	32 (32.0%)	
GNRI Change				**0.0168**				
None/Increase	69 (22.0%)	32 (17.7%)	37 (27.8%)					
Mild Decrease	145 (46.2%)	81 (44.7%)	64 (48.1%)					
Severe Decrease	100 (31.8%)	68 (37.6%)	32 (24.1%)					

Cancer stage: Early (stages 1 and 2) and Late (stages 3 and 4); GNRI: Geriatric Nutritional Risk Index; No Risk: *no nutritional risk*, GNRI > 98; Any Risk: *Any nutritional risk*, GNRI ≤ 98. GNRI Change: no change or increase: GNRI change > 0, mild decrease: −15 ≤ GNRI change < 0, and severe decrease: GNRI change < −15. Bold indicates significant values.

**Table 2:** Cox proportional hazards model comparison.

	Model 1	Model 2	Model 3
Variable of Interest	Change in GNRI Score *	Change in GNRI score *	Change in GNRI score *
Covariates	GNRI score at diagnosis * Stage * Sex Race Age	GNRI score at diagnosis * Stage *	Stratified: GNRI score at diagnosis Stage *
*p*-value	<0.0001	<0.0001	<0.0001
Proportional Hazards: Global	0.015	0.0054	0.0400
Proportional Hazards: Variables	Change in GNRI: 0.131 GNRI at diagnosis: 0.013 Stage: 0.053 Sex: 0.539 Race: 0.128 Age: 0.801	Change in GNRI: 0.1374 GNRI at diagnosis: 0.0130 Stage: 0.0524	Change in GNRI: 0.057 Stage: 0.111
AIC	2199.43	2194.38	1899.65
BIC	2223.09	2207.90	1909.79

Note: * Significant variables.

**Table 3:** Cox proportional hazards model results for model 3.

Variable	Subgroup	Hazard Ratio	95% Confidence Interval	*p*-Value
GNRI Change over time Ref. None/Increase	Mild Decrease	2.194	1.459–3.299	0.000159
	Severe Decrease	4.038	2.640–6.177	<0.0001
Cancer Stage Ref. Early	Late	1.947	1.478–2.565	<0.0001

## Data Availability

The data analyzed during the current study are available from the corresponding author upon reasonable request.

## Supplementary Materials

The following supporting information can be downloaded at: https://www.mdpi.com/article/10.3390/nu17030509/s1, Table S1. Comparative analysis of included and excluded patients. Table S2. Survival Analysis by Study Inclusion and Excluded Groups. Table S3. Cox Survival Analysis Results Analyzing Age and Study Inclusion for the Total Sample (N = 910). Figure S1. Comparison of survival based on inclusion in the final sample.
