# Supplementary material for: Geriatric Nutritional Risk Index (GNRI) and Survival in Pancreatic Cancer: A Retrospective Study
Source: Nutrients. 2025 Jan 30;17(3):509. doi: 10.3390/nu17030509 (PMC11819935; doi:10.3390/nu17030509)
Supplement: Supplementary file 1 [file nutrients-17-00509-s001.zip › nutrients-3401511-supplementary.pdf]

Supplementary Table S1. Comparative analysis of included and excluded patients				
Variable	Total Sample (n=910)	Included (n=314)	Excluded (n=610)	p-value
Survival (Days)				
Mean	1047.1	1093.8	1023.1	0.3004
Median	654.0	725	567.0	
Range	1 - 3164	6 - 3164	1 - 3152	
Age (Years)				
Mean	71.7	70.5	72.2	**0.03274**
Median	73.0	71.0	73.0	
Range	27 - 101	30 - 98	27 - 101	
Sex				
Male	501 (54.2%)	174 (55.4%)	327 (53.6%)	0.6508
Female	423 (45.8%)	140 (44.6%)	283 (46.4%)	
Race				
White	741 (80.2%)	263 (83.8%)	478 (78.4%)	0.0625
All other	183 (19.9%)	51 (16.2%)	132 (21.6%)	
Stage				
Early	480 (51.9%)	176 (56.1%)	304 (49.8%)	0.08517
Late	444 (48.1%)	138 (43.9%)	306 (51.2%)	
Note. Comparative analysis included t-test for continuous and chi square tests for categorical variables				

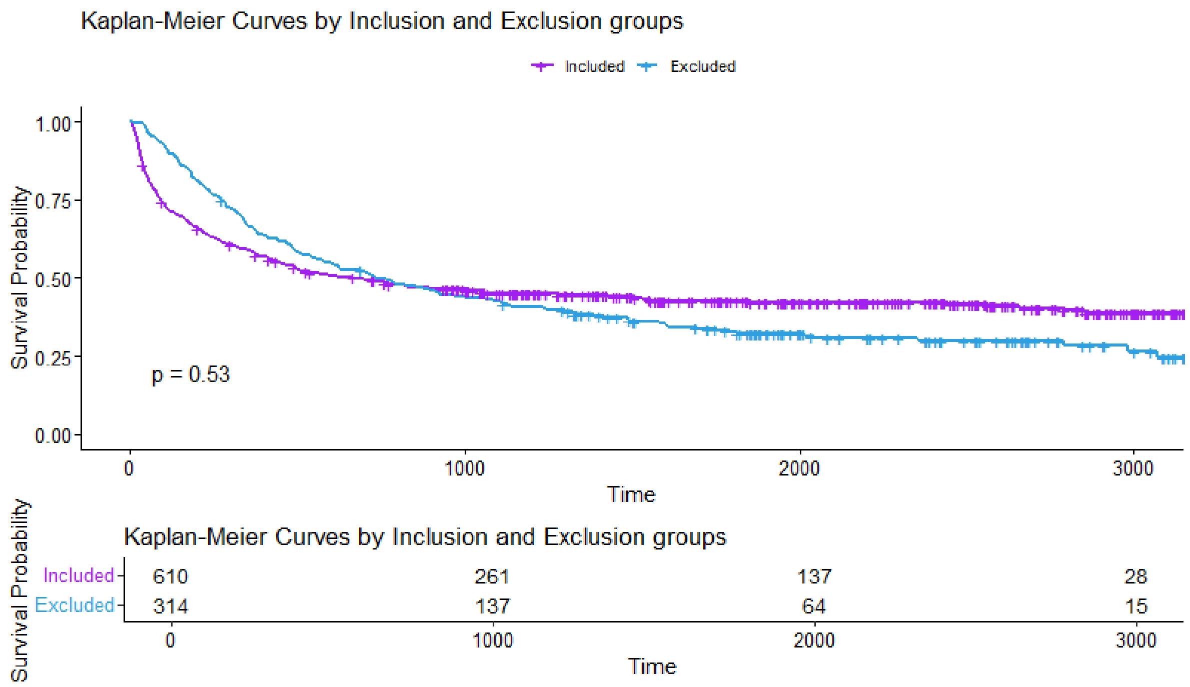

Supplementary Figure S1. Comparison of survival based on inclusion in the final sample

Supplementary Table S2. Survival Analysis by Study Inclusion and Excluded Groups			
Variable	p-value		
	Total Sample	Included	Excluded
Age	0.0092	0.222	0.0162
Sex	0.33	0.53	0.62
Race	0.68	0.86	0.68
Stage	<0.0001	<0.0001	<0.0001
Note. Survival analysis included Kaplan Meier tests for categorical variables and Cox regression Analysis for continuous variables.			

Supplementary Table S3. Cox Survival Analysis Results Analyzing Age and Study Inclusion for the Total Sample (N=910)			
Variable	Hazard Ratio	95% Confidence Interval	p-value
Age	1.0119	1.0025 – 1.020	0.0132
Inclusion Group	1.5584	0.5290 – 4.591	0.4209
Interaction	0.9948	0.9801 – 1.010	0.4896
